# Accuracy of procalcitonin for diagnosing sepsis in adult patients admitted to the emergency department: a systematic review and meta-analysis

**DOI:** 10.1186/s13643-023-02432-w

**Published:** 2024-01-22

**Authors:** Hany A. Zaki, Soumaya Bensliman, Khalid Bashir, Haris Iftikhar, Mohamed H. Fayed, Waleed Salem, Amr Elmoheen, Yavuz Yigit

**Affiliations:** 1 Hamad Medical Corporation Doha, Ar-Rayyan, Qatar; 2 Medicine, Qatar University, Doha, Qatar; 3 Blizard Institute, Queen Mary University, London, UK

## Abstract

**Background:**

Differentiating sepsis from non-infectious systemic inflammatory response syndrome (SIRS) is challenging. Biomarkers like procalcitonin (PCT) aid early risk assessment and guide antibiotic use. This study aims to ascertain PCT's accuracy as a sepsis biomarker among adult emergency department admissions.

**Method:**

The PRISMA guidelines were followed to search for relevant articles in five electronic databases between April 14th and August 4th, 2023: PubMed, Cochrane Library, ProQuest, EMBASEs, and ScienceDirect. Studies had to be published in English to avoid directly translating scientific terms. Besides, the inclusion criteria were based on the diagnosis of sepsis in adult patients admitted to an emergency department. QUADAS-2 tool provided by the Review Manager version 5.4.1 was utilized to assess the risk of bias in included studies. STATA (v. 16) software was used to perform the meta-analysis.

**Results:**

Ten of 2457 studies were included. We sampled 2980 adult sepsis patients for the under-investigated role of PCT in ED sepsis diagnosis. PCT emerged as the primary early diagnostic biomarker with high levels (29.3 ± 85.3 ng/mL) in sepsis patients. Heterogeneity in outcomes, possibly due to bias in cohort and observational studies, was observed.

**Conclusion:**

PCT tests offer moderate accuracy in diagnosing sepsis and stand out for rapidly and precisely distinguishing between viral and bacterial inflammations.

## Background

As a living organism, human immune systems have developed over time to respond to the invasions of infectious pathogens by employing a host of defense mechanisms. The systematic response by the human body to infectious microbial agents constituting fungi, yeast, and bacteria is called sepsis [1]. As an inflammatory response to microbial invasion, sepsis may result from infections commonly acquired from hospitals, communities, and other medical care facilities. Patients suffering from sepsis are characterized by the development of fever, tachypnea, leukocytosis, and tachycardia [1] as the most common signs and symptoms. In severe cases, sepsis is related to dysfunction and hypoperfusion of at least one organ. Besides, when severe sepsis is accompanied by multiple system organ failures or hypoperfusion, it culminates into a fatal condition called septic shock. For this reason, sepsis is a life-threatening illness associated with a mortality rate of 18 to 45% in patients who are critically ill from the condition [2]. In addition, there has been an ever-growing number of people suffering from the condition globally, with over 18 million new cases of sepsis being reported annually [3].

The high number of new sepsis infections in the world's critical care units, such as the emergency department (ED), has been accompanied by a significantly increased mortality rate of 30% to 50% [4]. For instance, in the United States alone, epidemiological research indicates that approximately 750,000 people are infected with sepsis yearly [4]. Conversely, 28.3% of patients are infected with sepsis during their ICU stay in India, which is related to high prevalence, accounting for a mortality rate of 34% of those contracting the disease [5]. In many parts of the world, the signs and symptoms of sepsis are influenced by several factors that result in high variability ranging from bioburden and virulence of the sepsis-causing pathogen-host susceptibility to the portal of entry into the system [6]. Besides, infectious microorganisms invade the human body through the bloodstream during sepsis, proliferating directly and locally into the system, releasing many virulent factors, and infecting the bloodstream. The infectious products may stimulate the release of defense mechanism responses in endogenous mediators of sepsis from monocytes, plasma cell precursors, endothelial cells, and macrophages. Therefore, in most scenarios, the sepsis-causing and associated inflammatory response arises when the human body attempts to intervene and neutralize the infectious pathogens in the bloodstream. As a result, various defense mechanisms are activated. The immune cells respond by secreting the inflammatory protein, resulting in tissue damage and escalating to organ failure of the pathogen-host.

Numerous efforts and progress have been made in clinical interventional guidelines that mediate sepsis treatment. The most common intervention sought is early identification of the condition, and recommended application of a broad spectrum of antibiotics is the primary form of treating sepsis. Sepsis must be identified as early as possible since a missed identification leads to delayed treatment, which escalates the condition increasing the risk of death. Although many acute respiratory tracts (ART) infections, including suspected cases of sepsis, are often treated by the initiation of empiric antibiotics [7], bacterial pathogens, on the other hand, are challenging to detect, along with viruses accounting for a considerable proportion of these ART illnesses. Similarly, this phenomenon is common in patients suffering from suspected sepsis or systematic inflammatory response syndrome, in which other viral diseases and inflammatory illnesses may be the causes of a large number of these cases.

Integrating a host response marker corresponding to the infectious bacteria in the medical care of patients presenting sepsis and other ART illnesses has improved the body’s antibiotic response. PCT is among the host-response and blood infection markers that have been effectively used [8]. Based on SEPSIS-3, PCT can support sepsis prognosis and predict mortality over other parameters under the revised sepsis definition [9]. However, as a precursor hormone of calcitonin, PCT cannot be detected in healthy people. Nevertheless, PCT productions are often upregulated to intervene in bacterial infections, rapidly decreasing with patient recovery. PCT has generated more attention, being approved for guidance in antimicrobial therapies due to its ability to correlate with bacterial infections [10] more than other markers, such as the count of WBC (white blood cells) and CRP. Additionally, PCT provides a more favorable kinetic profile with the pathogen making it ideal for response assessment to the treatment [10]. Furthermore, the application of PCT is evolving daily as an intervention for managing sepsis which is widely used as a diagnostic biomarker for sepsis and other bacterial infections.

Systematic reviews and meta-analyses have been carried out to compare PCT’s diagnostic accuracy to other biomarkers (presepsin and C-reactive) in assessing sepsis among adult and young patients [11–13]. As a result, this review focuses only on the diagnostic accuracy of PCT among adults (critically admitted in emergency departments) due to the severe symptoms caused by the disease, such as loss of consciousness, slurred speech, and variation in mental wellness. This article's primary objective is to evaluate PCT's role and accuracy as a host-response maker in diagnosing sepsis among patients in emergency departments. Determining the role of this biomarker in diagnostic pathways will help handle patients admitted to emergency departments. Besides, it will help design future research to examine the diagnostic accuracy of tests.

## Methods

### Protocol and registration

This systematic review and meta-analysis followed the commendations on preparing and reporting systematic reviews and meta-analyses pre-defined by the PRISMA (Preferred Reporting Items for Systematic Review and Meta-Analyses) statement and the Cochrane collaboration guidelines.

### Eligibility criteria

The study employed inclusion and exclusion criteria as the basis on which studies were identified, included, and excluded others from obtaining only the relevant and primary studies that explored the role of PCT in diagnosing sepsis in adult patients. Studies were included if they met the following criteria for inclusion.

1. Study design and type: reviewers included randomized controlled trials, cohort studies, observational, prospective, and retrospective studies. Studies must be published in English. The criterion was essential to avoid direct translation of scientific terms from other languages other than English, which could result in different meanings and interpretations. Also, they must be published between 2011 and 2022. The condition was vital to include recent developments involving sepsis diagnosis among adult patients.
2. Participants: we included studies that included adult patients as research subjects ($\ge$ 18 years) with severe sepsis, septic shock, or sepsis according to international consensus definitions admitted at the emergency department.
3. Intervention: we included research studies that conducted serum PCT assessment in one or more comparison groups
4. Comparison: reviewers expected comparison groups evaluating standard approaches for sepsis diagnosis and biomarkers other than PCT, such as interleukins and CRP.
5. Outcome: sensitivity, specificity, precision, and positive and negative likelihood ratio of PCT and comparison intervention methods.

On the other hand, studies were excluded based on the following conditions.

1. Study design: reviewers excluded secondary studies such as meta-analyses, systematic reviews, case reports on PCT and sepsis, and studies published before 2011
2. Outcome: Studies with unrelated intended outcomes that we did not need for this research. Also, we excluded studies that had incomplete results or had apparent mistakes that could negatively impact our meta-analysis

The screening of studies was performed independently by two review authors to establish if the studies met the eligibility criteria. The discrepancies during the screening process were solved through consultation with the third reviewer.

### Information sources and search strategy

Only an electronic database search was performed for this systematic review and meta-analysis of relevant studies. A comprehensive search strategy was employed in navigating several electronic databases per guidelines stipulated by PRISMA. Between April 14th and August 4th, 2023, the electronic databases explored and utilized during the search included Science Direct, PubMed, Cochrane Library, ProQuest, and EMBASE. The most recent search was performed on August 2023. For study in this particular review, search studies dating from 2011 to 2022 were included in the research. During the search process, a database search was conducted based on keywords and headings that appeared in the text of the study. For effective search, keywords and terms such as “procalcitonin,” or “PCT,” or “sepsis,” and “procalcitonin diagnosis” were used to search and navigate the database. To filter out specific studies, terms like “the role of procalcitonin,” “sepsis diagnosis in adult patients,” and “PCT diagnosis of sepsis” were utilized. Two research investigators searched the five databases for the appropriate literature independently. The third author was consulted to solve any discrepancies during the literature search. A detailed search strategy for each database is shown in Appendix 1.

### Quality assessment

Two review authors performed the risk of bias assessment via the Quality Assessment of Diagnostic Accuracy Studies (QUADAS-2) tool provided by the Review Manager software (RevMan 5.4.1). The tool comprises four assessment domains: patient/participant selection, reference standard, index standard, flow, and timing. Three fields utilized for the applicability include index test, reference standard, and patient selection.

### Data extraction

Data extraction was assigned to two investigators who independently reviewed and analyzed the included studies per the PICO guidelines. The discrepancies between the two reviewers were solved through discussions and consultation with other review authors until a consensus was reached. The reviewers’ data focused on PCT's role in diagnosing sepsis in adult patients. The relevant data retrieved by the reviewers from the studies encompassed; study ID (author(s) and year of publication), participants (age), sample size, study design, intervention, and outcomes (sensitivity, specificity, diagnostic odds ratio, and diagnostic positive and negative likelihood ration).

### Statistical analysis

This systematic review undertook an initial descriptive analysis of the included studies. The random effects model analyzed the pooled specificity and sensitivity, diagnostic odds ratio, and positive and negative predictive values. Heterogeneity between these studies was evaluated using the inverse variance (*I*^2^) statistic, illustrating the variation percentage but not sampling error across the studies. Higgins shows that any *I*^2^ value above 75.0% demonstrates high heterogeneity [14]. Meta-analysis was performed using the STATA (v. 16) software. A 95% confidence interval (CI) was used to calculate PCT’s pooled sensitivity and specificity in diagnosing sepsis.

## Results

### Search results

Figure 1

**Fig. 1:**
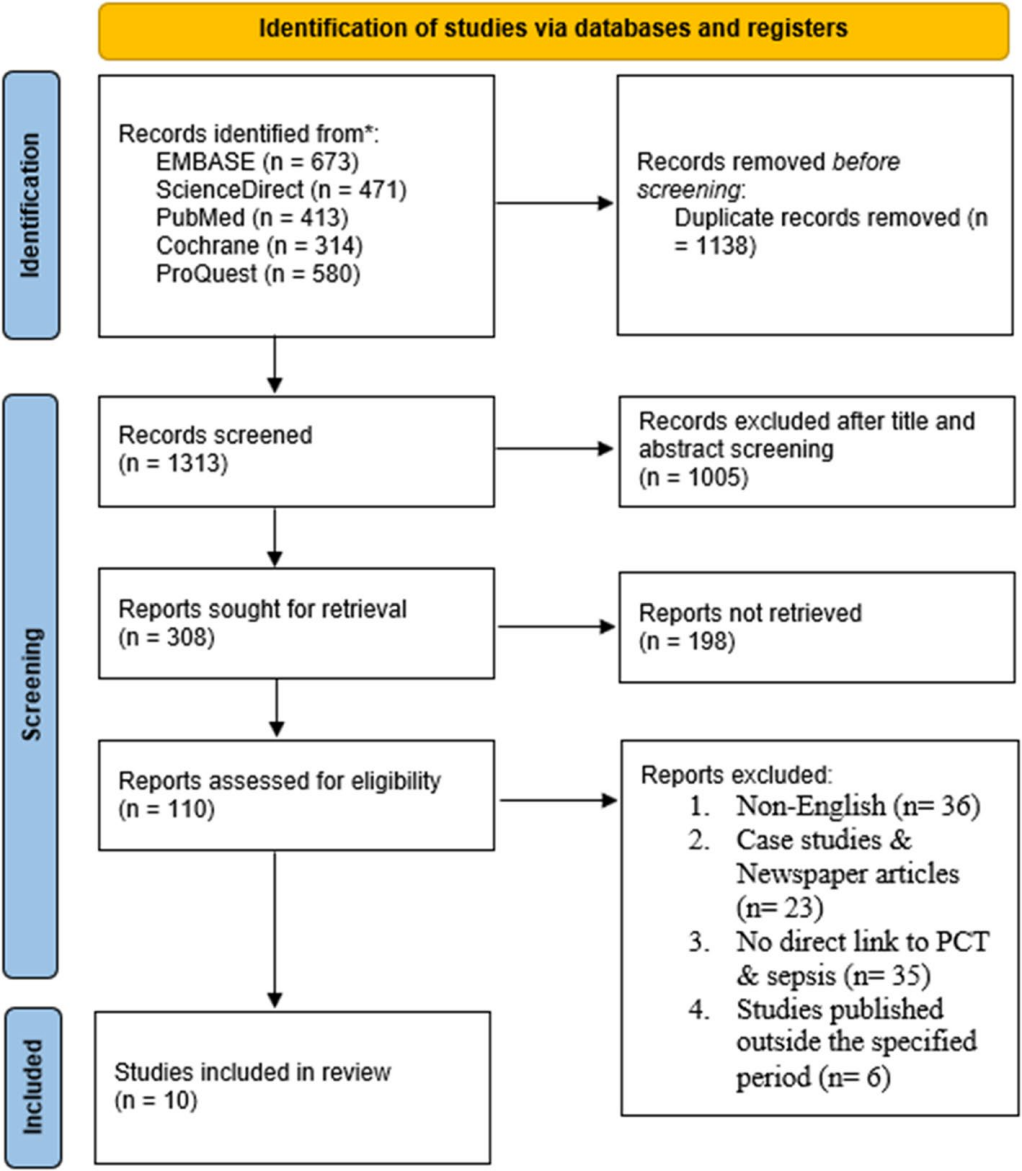
PRISMA flow chart detailing the search strategy

### Study characteristics

In the analysis of the studies on PCT's role in the diagnosis of sepsis, three studies compared PCT's role and utility to other biomarkers, such as lactate [15], presepsin [16], and mid-regional pro-Adrenomedullin (MR-proADM) [10], in the diagnosis of sepsis in the emergency department. Four studies focused on the diagnostic value and the utilization of serum PCT [17], C-reactive protein (CRP) [18], serum lactate [19], and Il-6 in predicting bacteremia in adult patients [20] and diagnosis of bacterial sepsis.

Besides, one study focused on the association of blood cultures to PCT levels in patients with septic shock in the emergency department [21]. In addition, one study focused on PCT as an early indicator of progressive septic shock in sepsis patients associated with ureteral calculi at the emergency department (ED) [22]. One study focused entirely on PCT as an inflammatory biomarker for suspected cases of sepsis [23]. Out of 2980 adult patients sampled as study participants, only 2162 (72.6%) patients met the inclusion criteria in all ten studies included in this systematic review (Table 1).

**Table 1:** Characteristics of included studies

Author ID	Study design	Participants	Intervention	Outcomes
Miglietta et al. (2015) [19]	Retrospective study	One hundred forty-five critically ill patients with a mean age of 60	Utilizing PCT, CRP, and lactate in diagnosing SIRs, systemic candidiasis, and bacterial sepsis	Significantly higher levels of PCT were associated with *Staphylococcus aureus* at zero (0) days compared to other Gram-positive species. Besides, the PCT levels were significantly elevated in Gram-negative sepsis relative to the sepsis associated with Gram-positive bacteria. However, the *P* value was not much significant Diagnostic sensitivity and specificity for PCT and CRP between bacterial sepsis and SIRS of sepsis were 85.7% and 83.3%, respectively. The positive and negative predictive values were 89.6% and 77.7%, respectively
Nakajima et al. (2014) [17]	Prospective study	Forty-five patients with sepsis comprised 29 male and 16 female patients of the age bracket of 57.2 ± 17.7 years old. Twenty-four pneumonia patients (15 men and nine women) of 70.5 ± 9.8 years old, with 56 control patients constituting 13 women and 43 men of age 62 ± 18.3 years	Clinical utilization of PCT as a marker of sepsis	The levels of PCT in the sepsis group showed statistically significant differences among the three groups. In the sepsis group, the PCT level was recorded as 29.3 ± 85.3 ng/mL, which was significantly higher than the PN levels (0.34 ± 8.6 ng/mL). In the control group, the level was 0.74 ± 2.1 ng/mL The positive and negative predictive values for PCT were 85.7% and 51.6%, respectively. On the other hand, PCT sensitivity and specificity were 57.6% and 87.5%, respectively
Oliveira et al. (2013) [18]	Randomized clinical trial	Three hundred fifty-five adult patients of age 18 years were assessed for inclusion. Of these, 97 patients were randomized after excluding three, with 49 allocated to the PCT group and 45 to the CRP group. The mean age of the patients was 59.8 years, with a standard deviation of 16.8	In a randomized clinical trial, PCT and CRP guided antibiotic treatment and therapy in sepsis	The duration for the first episode of antibiotic therapy provided a similar result, with a 7-day median associated with the PCT group and a 6-day median for the CRP group. The antibiotic therapy number of days was more significant in the PCT group than in the CRP group during the follow-up period
Kece et al. (2016) [15]	Prospective study	Out of the 94 eligible patients, 86 cancer patients with a median age of 61 years	Comparison of the utility of lactate and Procalcitonin (PCT) levels in the diagnosis of sepsis in cancer patients	Concerning the diagnosis of sepsis, 0.8 ng/mL of PCT provided a specificity of 76.56% and a 63.64% sensitivity which was associated with a positive 2.72 LR with a 0.47 negative LR. The procalcitonin values alone cannot be suggested to distinguish sepsis from other non-infectious SIRS in adult patients with cancer to guide decisions made in the emergency department PCT sensitivity and specificity were 63.64% and 76.56%, respectively, whereas the positive and negative predictive values were 2.72 and 0.47, respectively
Ulla et al. (2013) [16]	Prospective study	189 patients of 18 years and above	PCT was compared with presepsin in the emergency department’s diagnostic and prognostic management of sepsis	PCT was associated with higher diagnosis accuracies with areas under the curve (AUCs) of 0.875 compared to 0.701 presepsin. Septic patients had a high presepsin concentration, with a similar trend for mean PCT values PCT was associated with higher sensitivity (89.47%) and specificity (75.90%)
Ko et al. (2016) [22]	Retrospective study	Among 574 patients identified for assessment, 49 were aged 24 to 88 (mean age of 67.2)	PCT was determined as an early indicator of progressive septic shock in sepsis patients associated with ureteral calculi at the emergency department (ED)	PCT is superior as a biomarker in diagnosing sepsis patients and predicting those at high risk of progressing septic shock in patients with urolithiasis The sensitivity and specificity were 86.70% and 85.30%, respectively, for PCT diagnosis of sepsis among patients
Lin et al. (2017) [20]	Retrospective study	886 patents	PCT, lactate, high sensitivity CRP in bacteremia prediction of adult patients admitted in ED	PCT was associated with 0.72 for positive blood culture, with a 3.9 ng/mL derived opposite cutoff. Lactate was related to 0.6 with a corresponding 17.9 optimal cutoff. In contrast, CRP was 0.56 with 13 mg/dL optimal cutoff. PCT’s diagnostic odds ratio was 3.54 (95% CI 2.46–5.51), while the sensitivity and specificity were 81.0% and 47.0%, respectively. Similarly, PCT displayed 1.51 positive predictive value and 0.41 negative predictive value
Webb et al. (2020) [21]	Cohort study	148 patients of age bracket between 19 to 98 years with a median age of 72	PCT level related to positive blood culture (BC), septic shock, and in-hospital mortality associated with septic patients in ED	A 0.58 ng/mL corresponded to the median initial PCT with a 0.16–5.36 IQR. The median maximum of PCT was recorded at 2.1 ng/mL with a corresponding 0.3–11.1 IQR. Patients with negative BC had a median maximum PCT of 1.06 ng/mL compared to their positive BC counterparts with 4.19 ng/mL. PCT’s diagnostic odds ratio was 3.35 (95% CI 1.67–6.70)
Travaglino et al. (2012) [10]	Observational multicentric study	128 patients aged 18 years and above	PCT and Mid-regional pro-Adrenomedullin (MR-proADM) utility in ill febrile patients in ED, APACHE II comparison	The MR-proADM control values ranged between 0.4 to 0.58 nmol/L relative to 0.5–1.68 nmol/L in patients. Control PCT values ranged from 0.04 to 0.008 ng/mL against 0.1 to 3.4 ng/mL in patients. Patients admitted complaining of fever had 0.694 AUC for MR-proADM, while PCT was associated with an AUC of 0.763, with the combined use of both PCT and MR-proADM indicating an AUC of 0.79
Tsalik et al. (2012) [23]	Cohort study	336 patients	Inflammatory biomarkers value for suspected cases of sepsis	There is a high relationship between PCT and various sepsis-associated outcomes, such as the likelihood and severity of infection and septicemia. Similarly, both CRP and IL-6 were also associated with similar sepsis-related results. PCT’s sensitivity and specificity in sepsis diagnosis were 40.70% and 87.20%, respectively. Positive and negative predictive values for PCT sepsis diagnosis were 89.80% and 34.60%, respectively

### Risk of bias assessment

**Fig. 2:**
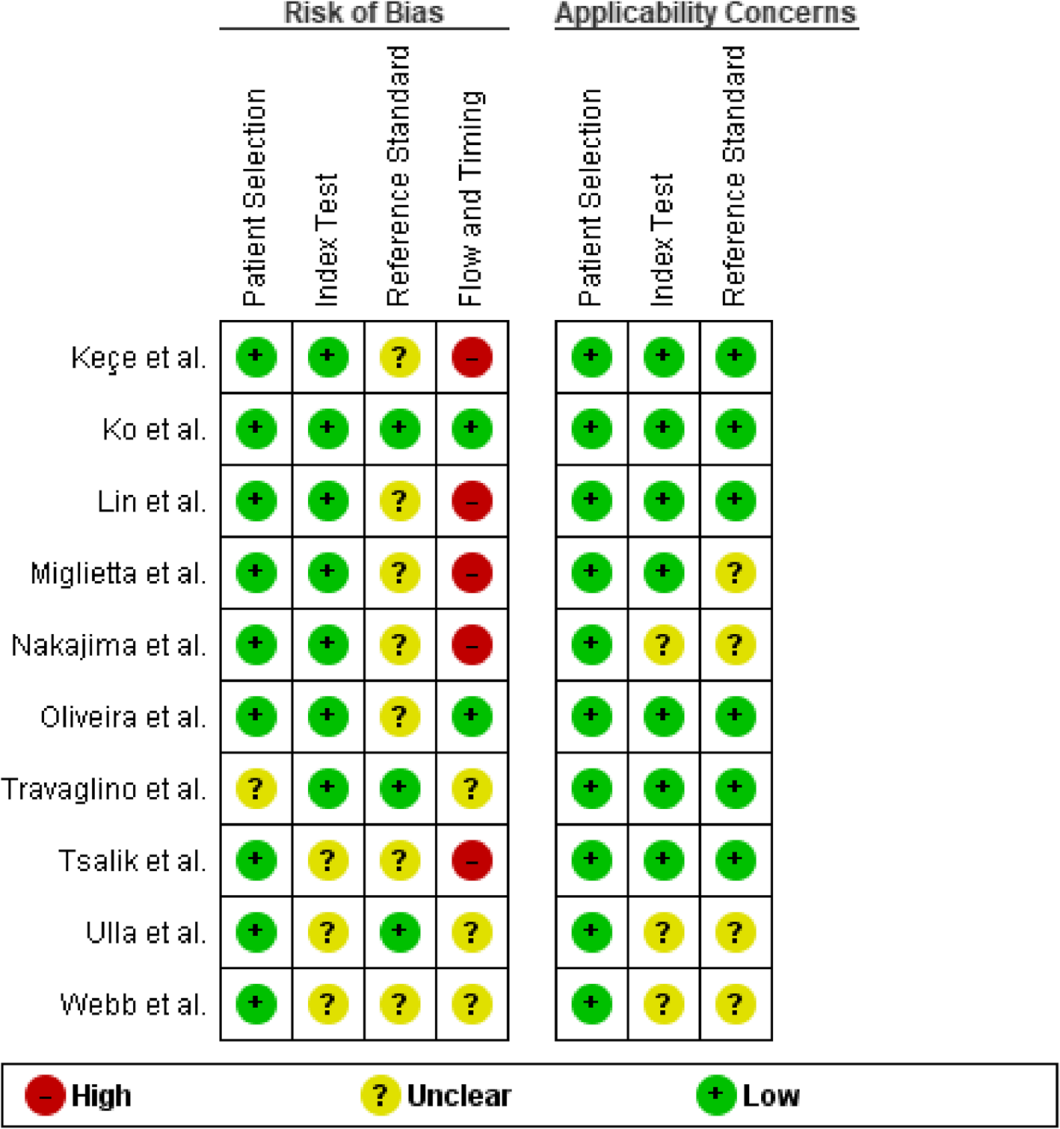
QUADAS-2 Risk of bias and applicability concerns summary: review authors’ judgments about each risk of bias item presented as percentages across all included studies

The outcomes of the assessment of bias risks conducted using the QUADAS-2 tool are provided in Fig. 2 below. Studies that illustrated high or unclear index risk owing to the absence o or lack of explicit pre-specified cutoff PCT threshold for sepsis diagnosis. High risks are represented by a red-colored circle, low risks by a green circle, and a yellow circle for unclear risks. Uncertainties and unclear risks imply insufficiently and lack of clear judgment associated with the studies' little details.

### Diagnostic accuracy of PCT

#### Sensitivity and specificity

**Fig. 3:**
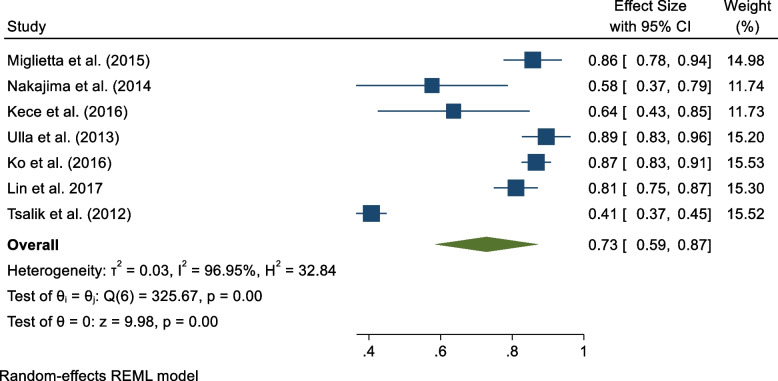
Sensitivity of PCT in sepsis diagnosis

**Fig. 4:**
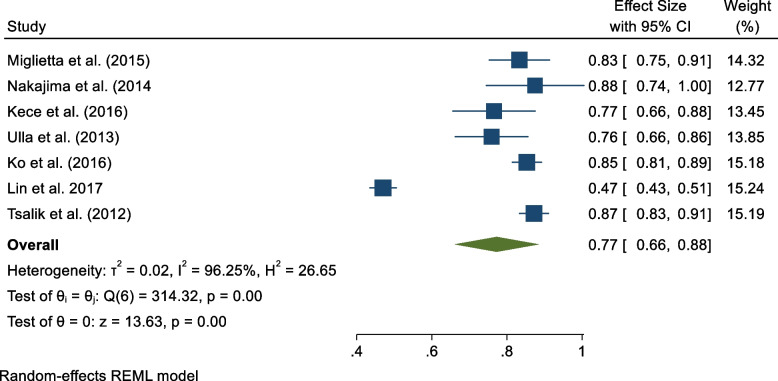
Specificity of PCT in sepsis diagnosis

Results from seven studies were pooled for sensitivity and specificity analysis [15–17, 19, 20, 22, 23]. Figures 3 and 4 are forest plots that show the sensitivity and specificity ranges for PCT in sepsis diagnosis. Data pooled from six studies resulted in sensitivity and specificity of 0.73 (95% CI 0.59 to 0.87) and 0.77 (95% CI 0.66 to 0.88) for diagnosing sepsis among adult patients. However, the study outcomes for sensitivity and specificity had considerable heterogeneity (${I}^{2}$= 96.95% and ${I}^{2}$= 96.25%, respectively).

### Diagnostic odds ratio

**Fig. 5:**
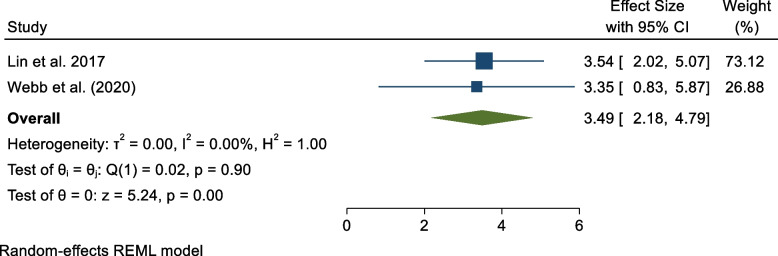
PCT's diagnostic odds ratio

Two studies [20, 21] reported PCT’s diagnostic odds ratio in sepsis diagnosis. The pooled evidence shows that PCT had a diagnostic ratio of 3.49 (95% CI 2.18, 4.79) (Fig. 5).

### PCT positive and negative predictive values

**Fig. 6:**
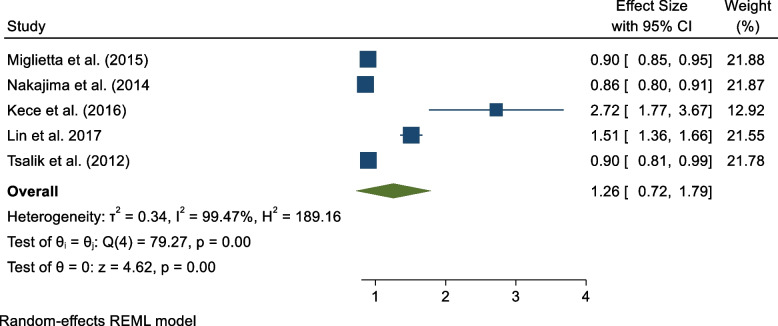
PCT's positive predictive value in sepsis diagnosis

**Fig. 7:**
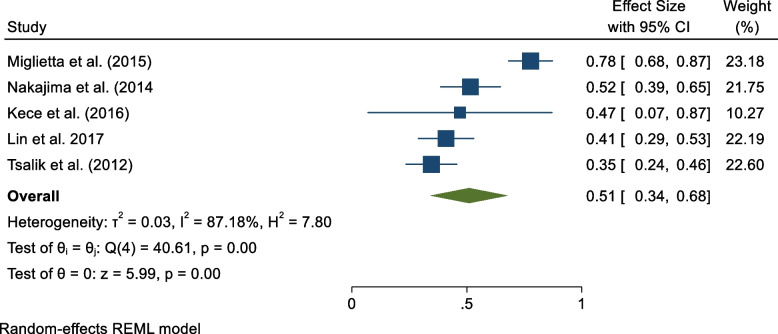
PCT’s negative predictive value in sepsis diagnosis

Five studies reported PCT's negative and positive predictive values in diagnosing sepsis among patients [15, 17, 19, 20, 23]. From the pooled results, PCT positive and predictive values were 1.26 (95% CI 0.72, 1.79) and 0.51 (95% CI 0.34, 0.68), respectively (Figs. 6 and 7).

**Discussion**

Sepsis has proved to be the primary source of death for critically ill adult patients in the emergency department [22]. Therefore, conducting an early diagnosis of sepsis for the following resultant treatment to improve the outcomes is becoming essential. Since the clinical signs and symptoms associated with sepsis usually overlap with other SIRS (systemic inflammatory response syndrome) [24], there is a need for an effective biomarker to facilitate the identification and distinguish sepsis from other non-infectious SIRS [23]. The diagnosis of sepsis encompasses some degree of host response whose manifestation forms the basis for correctly administering antimicrobial agents. Nevertheless, in adult patients with neonate and immunosuppressed characteristics, the representation is unlikely to present, which makes these patients susceptible to sepsis infections. Therefore, the ideal biomarker for bacterial infection should be capable of detecting the presence of SIR infections in patients without causing host response with high sensitivity and specificity enough to distinguish infection from other induced SIRS.

Research shows that various clinical and biochemical makers have had poor specificity and sensitivity when predicting early bacteremia in febrile-ill patients in ED [25–27]. Rapid detection and diagnosis of bacterial infections facilitate early implementation of antimicrobial therapy, thereby identifying the septic patients at high risk for complications [24]. Moreover, discriminating and properly distinguishing bacterial sepsis in many cases of febrile ill patients provided significant advantages of reduction in hospitalization and antimicrobial usage, facilitating clinical focus and offering alternative diagnosis pathways [10, 28]. In this review, PCT has been identified as the primary candidate biochemical marker widely employed in the early diagnosis of sepsis. One of the studies included in this review found that the levels of PCT (29.3 ± 85.3 ng/mL) were significantly high in sepsis patients compared to the classified control group of patients (0.34 ± 8.6 ng/mL) [17]. Besides, from the ROC analysis, the levels of PCT indicated an area under the curve (AUC) of 0.80, which suggested that employing the PCT test provided moderate accuracy in sepsis diagnosis [16]. Similarly, previous studies also investigated the role of engaging PCT as a positive predictor biomarker for sepsis, with various results associated with its clinical suitability [25–27].

Furthermore, PCT remains the biomarker for bacterial sepsis following its ability to indicate high levels of efficacy in sepsis diagnosis. Research by Brodská et al. suggested that higher levels of PCT significantly differentiate gram-positive sepsis from gram-negative sepsis, providing alienation from fungemia [29]. Miglietta et al. indicate that PCT levels, determined at day zero on the onset of the symptoms and signs associated with sepsis, were considerably higher in Gram-negative sepsis than those induced by Gram-positive bacteria [19]. However, the study did not indicate any statistical disparity, with a *P* value associated with the two subgroups insignificantly different (*P* = 0.096). *S. aureus* sepsis reflected a similar generalized PCT level to Gram-negative sepsis [19]. Nevertheless, the study indicated that PCT is not a superior discriminative biochemical maker among differing bacterial infections in the bloodstream, which contrasts with the results from Brodská et al.’s study [29]. On the other hand, dramatically high PCT levels are often related to Gram-negative sepsis (above 20 ng/ml). In comparison, infrequent levels above 20 ng/ml were recorded among gram-positive sepsis, despite being exclusive in *Staphylococcus aureus* [19].

The study by Webb et al. shows that the levels of PCT were dramatically high in sepsis patients that tested positive for blood culture [21]. Nakamura et al.’s study indicated that blood culture (BC) has proved to be the primary clinical index for sepsis, whose final diagnosis can be derived from a positive BC [30]. However, the use of blood culture is usually associated with a long period of completing the process of sepsis diagnosis. On the contrary, employing PCT facilitates the quick performance of the process, with the associated acceptable span of PCT adjustment being more prolonged than that indicated by BC. From the Wilcoxon estimate of the discriminative ability of sepsis patients with optimistic blood cultures prediction, PCT levels were at 0.72, corresponding to 95% CI [0.69–0.75] at 3.9 ng/mL best cutoff. In predicting the positive blood culture, PCT’s diagnostic odds ratio (OR) was recorded at 3.64 (95% CI [2.46–5.51]).

Based on these estimates, the ROC comparisons indicated a statistically significant difference between the discriminative abilities of PCT (*P* < 0.001), lactate (*P* < 0.001), and CRP [20]. The high PCT levels in patients that indicated positive blood cultures confirm the PCT assay’s role in diagnosing bacterial sepsis and infections [31]. Moreover, for the septic adult patients in the emergency department, the study found that PCT has the capabilities to discriminate [21] and distinguish bacterial blood cultures [32] with better abilities associated with Gram-negative bacteremia [20]. Such capabilities are better than other biomarkers, such as CRP and serum lactate, which contradicts Keçe et al.’s study [15].

According to Christ-Crain et al.’s ROC analysis, the diagnosis of bacteremia indicated that PCT levels are comparably a better biomarker than CRP [33]. The appropriate cutoff value of 0.38 μg/L for PCT was associated with high NPV, while a high PPV was related to 0.83 μg/L [33]. Based on these findings, the author concluded that antibiotic therapy conducted under the guidance of PCT is encouraged when the level of PCT is more significant than 0.25 μg/L and much more inspired when the PCT level is more than 0.5 μg/L. On the contrary, this systematic review and meta-analysis's findings contradict this study [18]. Employing a PCT-based protocol for patients with sepsis in the intensive care unit (ICU) was not superior to the CRP-based protocol in guiding antibiotic therapy treatment. On the contrary, PCT has been deemed an excellent biomarker in diagnosing sepsis patients and predicting those at high risk of progressing to septic shock in patients with urolithiasis [22]. However, Keçe et al. highlight that PCT cannot be used alone in distinguishing sepsis from non-infectious SIRS in adult septic patients [15].

Antibiotic therapy’s duration of the first infection episode was observed to show similar outcomes for both CRP and PCT groups. The PCT group was associated with 7.0 days median (Q1–Q3, 6.0–8.5), which was not significantly different from the CRP group with a 6.0 days median (Q1–Q3, 5.0–7.0) in the CRP group [18]. Furthermore, after the severity adjustment using the APACHE II, the results indicated have remained the same with a resultant HR of 1.206 (95% CI, 0.774–1.3; *p* = 0.13). Nevertheless, during the antibiotic therapy, the total number of days associated with the PCT group was more significant than those in the CRP group. However, the difference between the two groups indicated by thirteen and eight days was insignificant. Another study conducted by a French multicenter study with 630 participants comprising critically ill adult septic patients showed that the antibiotic-free period was substantially higher with the PCT-guided antibiotic group (14.2 ± 9.1 days) relative to routinely conducted criteria 11.6 ± 8.2 days [34].

## Limitations of the study

This review acknowledges several limitations that warrant consideration. Firstly, the scope of the review was confined to studies published between 2011 and 2022, thus excluding prior research, potentially omitting crucial insights that could have enriched this systematic review and meta-analysis. Moreover, the review primarily emphasized the role of Procalcitonin (PCT) in sepsis diagnosis, a deliberate focus aimed at mitigating bias. However, this deliberate choice resulted in the exclusion of studies investigating alternative biomarkers, potentially limiting the comprehensive understanding of sepsis diagnosis.

It's important to note that due caution is advised when extrapolating the findings of this review, given the limited pool of studies focusing on PCT’s diagnostic utility in septic patients. Additionally, the inclusion of observational case design studies introduced challenges in discerning PCT’s precise diagnostic contribution for sepsis among adult patients admitted to the emergency department with varying underlying conditions.

Furthermore, the potential for reporting bias cannot be discounted, as the inclusion of low-risk patients in some of the analyzed studies may have inadvertently skewed the overall findings. This selection bias could impact the comparative analysis of the diverse studies and potentially influence the review’s conclusions.

Lastly, a noteworthy concern stems from the heterogeneity in participant numbers across the ten studies incorporated into this review. This variation in sample sizes posed challenges in harmonizing and establishing robust correlations regarding the application of PCT as a diagnostic biomarker for sepsis among adult patients in the emergency department. These limitations collectively underscore the need for interpretive caution and context-awareness when interpreting the implications of this systematic review and its implications for clinical practice.

## Conclusion

As an essential biomarker, PCT is extensively applied in the medical field, particularly in diagnosing sepsis patients, compared to conventional biochemical sepsis markers. PCT has improved other traditional biomarkers, including CRP, in critically ill patients. However, based on this systematic review, meta-analysis findings, and other previously conducted studies, PCT may not be sufficient to act alone to diagnose invasive bacterial infection and its associated sepsis severity. PCT can be used better in ruling out as opposed to the ruling in the diagnosis of systemic sepsis in a critical care context such as the emergency department, primarily when repeated evaluations are employed. Therefore, recommendations are made for combining two or more biomarkers as a more effective clinical diagnosis of sepsis. However, much investigation and research are needed to establish the ideal tool for clinical applications by combining biomarkers.

## Data Availability

The datasets used and/or analyzed during the current study are available from the corresponding author upon reasonable request.

## Appendix

### Detailed search terms

#### PubMed

("procalcitonin"[Supplementary Concept] OR "procalcitonin"[All Fields] OR "procalcitonin"[MeSH Terms]) OR PCT[All Fields] OR (PCT[All Fields] AND ("diagnosis"[Subheading] OR "diagnosis"[All Fields] OR "diagnosis"[MeSH Terms]) AND ("sepsis"[MeSH Terms] OR "sepsis"[All Fields]) AND ("emergency service, hospital"[MeSH Terms] OR ("emergency"[All Fields] AND "service"[All Fields] AND "hospital"[All Fields]) OR "hospital emergency service"[All Fields] OR ("emergency"[All Fields] AND "department"[All Fields]) OR "emergency department"[All Fields]) AND ("adult"[MeSH Terms] OR "adult"[All Fields] OR "adults"[All Fields])).

#### EMBASE

("procalcitonin" OR "procalcitonin" OR "procalcitonin" OR PCT AND ("diagnosis") AND ("sepsis") AND ("emergency service, hospital" OR ("emergency" AND "service" AND "hospital") OR "hospital emergency service" OR "department") OR "emergency department") AND ("OR "adults")).

#### Cochrane Library

("procalcitonin"[Supplementary Concept] OR "procalcitonin"[All Fields] OR "procalcitonin"[MeSH Terms]) OR PCT OR "diagnosis"[All Fields] OR "diagnosis"[MeSH descriptor]) AND ("sepsis"[MeSH Terms] OR "sepsis"[All Fields]) AND ("emergency service, hospital"[MeSH Terms] OR ("emergency"[All Fields] AND " AND "department"[All Fields]) OR "emergency department"[All Fields]) AND ("adult"[MeSH Terms] OR "adult"[All Fields] OR "adults"[All Fields])).

#### ScienceDirect

("procalcitonin" OR "procalcitonin" OR "procalcitonin" OR PCT AND ("diagnosis") AND ("sepsis") AND ("emergency service, hospital" OR ("emergency" AND "service" AND "hospital") OR "hospital emergency service" OR "department") OR "emergency department") AND ("OR "adults")).

#### ProQuest

Abstract(procalcitonin) AND sepsis AND summary(diagnosis) OR (emergency department) AND full text (emergency) AND (procalcitonin diagnosis).
